# Comparison of the effect of tildipirosin administered alone or in combination with transdermal flunixin on the performance, health, activity, and well-being of transported feedlot calves on arrival at the feedlot

**DOI:** 10.1093/tas/txaa005

**Published:** 2020-01-13

**Authors:** Miriam Martin, Michael Kleinhenz, Katie Kleinhenz, Emily Reppert, Dale Blasi, Timothy Parks, Angela Baysinger, John Hutcheson, Johann Coetzee

**Affiliations:** 1 Department of Anatomy and Physiology, Kansas State University College of Veterinary Medicine, Manhattan, KS; 2 Department of Clinical Sciences, Kansas State University College of Veterinary Medicine, Manhattan, KS; 3 Department of Animal Science, Kansas State University, Manhattan, KS; 4 Merck Animal Health, Kenilworth, NJ

**Keywords:** anti-inflammatory, cattle, drug, nonsteroidal, stress

## Abstract

Long distance transportation can be a significant source of stress to cattle and is associated with increased risk of bovine respiratory disease (BRD). The administration of a nonsteroidal anti-inflammatory drug (NSAID) has been shown to reduce stress following long distance transport. The objective of this study was to compare performance, health, accelerometer activity, and well-being between calves receiving either tildipirosin (Zuprevo 18%; Merck Animal Health, Madison, NJ) alone or in combination with transdermal flunixin (BANAMINE Transdermal Pour-on Solution; Merck Animal Health, Madison, NJ) on arrival at the feedlot. Three hundred eighty-four polled, Continental × English, and English crossbred bulls (*n* = 199) and steers (*n* = 185) were enrolled into one of two treatments: 1) tildipirosin administered in the neck as a single dose of 4 mg/kg only (PLBO) 2) tildipirosin in combination with transdermal flunixin applied to the dorsal midline at a dose of 3.3 mg/kg (FTD). Outcomes measured were average daily gain (ADG), dry mater intake (DMI), gain to feed, morbidity, mortality, accelerometer activity data, and a daily visual analog scale (VAS) assessment of well-being. Body weight (BW) was determined by weighing individual animals; ADG was calculated as initial BW—final BW / total days on feed; DMI was calculated as daily pen feed allocation—feed remaining at next feeding / number of calves in the pen; and gain to feed was calculated as pen level ADG / pen level DMI. The VAS used was a 100 mm line anchored at each end by descriptors of “no pain” or “severe pain”. Statistical analysis was performed using JMP 13 computer software using pen as the experimental unit, lot number as a random variable, and treatment as a fixed variable. There was no treatment effect on DMI (*P* = 0.51). During the first 14 d on feed, FTD calves had a lower ADG of 0.90 kg/d compared with 1.33 kg/d in the PLBO group (*P* = 0.05). There were no differences observed in morbidity and mortality between groups (*P* = 0.29). There were no treatment differences from activity data (*P* = 0.19). The VAS assessment showed a significant time × treatment interaction (*P* < 0.001). During the first 36 h after treatment administration, the FTD-treated calves had lower VAS scores [6.23 (95% CI: 5.27–7.20) compared with 7.28 (95% CI: 6.32–8.24)] than PLBO (*P* < 0.05). Results suggest that FTD-treated calves showed less signs of pain the first 36 h postdrug application relative to PLBO calves.

## INTRODUCTION

Negative effects of transporting calves are often a result of compounded stress due to weaning, social regrouping, and exposure to new pathogens (Fike and Spire, 2006). A strategy to reduce the acute-phase protein response elicited by transportation is to provide anti-inflammatory agents. Van Engen et al. (2014) previously demonstrated that administration of the nonsteroidal anti-inflammatory drug (NSAID) meloxicam PO at 1 mg/kg, reduced stress in calves following long distance transportation. Similarly, Cooke et al. (2013) demonstrated that flunixin meglumine administered intravenously at 1.1 mg/kg reduced the cortisol and acute-phase protein response elicited by road transport.

The incidence of bovine respiratory disease (BRD) is commonly associated with these stressors and is likely linked to changes in immune cell function and number. Inflammation from BRD within the lungs has a significantly negative affect upon performance parameters (Gifford et al., 2012). Most approaches to manage BRD are limited to vaccination and antibiotic use to decrease disease prevalence and severity (Penny, 2015). Developing strategies for physiological biomarker identification, treatment methods, and predictive behaviors may help reduce BRD incidence (Van Engen and Coetzee, 2018).

Flunixin transdermal has been effective in reducing prostaglandin E_2_ concentrations. Studies suggest that the anti-inflammatory effects of topical flunixin may last up to 48 h (Thiry et al., 2017). When flunixin is administered topically it is rapidly absorbed and has a longer half-life relative to intravenous administration (Kleinhenz et al., 2016). The impact of the co-administration of flunixin meglumine transdermal with an antimicrobial on arrival has not been investigated. If co-administration of flunixin transdermal with tildipirosin would improve the health and performance of high-risk calves on arrival at the feedlot, this would be beneficial to producers and veterinarians. The objective of this study was to compare performance, health, accelerometer activity, and visual assessment of well-being between calves receiving either tildipirosin alone or in combination with flunixin meglumine transdermal at 3.3 mg/kg on arrival at the feedlot.

## MATERIALS AND METHODS

### Animals, Housing, and Treatments

The Institutional Animal Care and Use Committee of Kansas State University reviewed and approved the experimental protocol for this project (IACUC# 4002).

This study was conducted at the Kansas State University Stocker Unit near Manhattan, KS, between October 2017 and January 2018. Calves were assembled through market facilities in Tennessee and shipped 12 h to the Kansas State University Stocker Unit via four different truckloads over a 10-d period. Calves remained assigned to four lots respective of each truckload throughout the study. A total of 397 polled, Continental × English and English crossbred bulls and steers were received for potential enrollment into the study. One hundred ninety-nine bulls and 185 steers were enrolled in the study, totaling 384 calves weighing an average of 218 kg. Calves displaying signs of illness or cryptorchidism were not enrolled. The study lasted 63 d from when calves arrived at the study site.

Upon arrival, calves were individually weighed and received pretreatment examinations which included; rectal temperature taken; ear notched for bovine viral diarrhea (BVDv) persistent infection testing (IDEXX Laboratories, Inc., Westbrook, ME); examined for health and physical abnormalities; and given an ear tag for visual identification and electronic identification (EID).

Calves were housed in 32 outdoor pens with dirt flooring of equal size (9.1 m × 15.2 m) with 12 calves/pen. Diets were formulated to provide 1.32 Mcal NEg/kg DM and offered at 2.2% BW. All treatments were fed once daily at approximately 0700 and refusals were collected and weighed daily before feeding. The diet was be formulated to contain 40% Sweet Bran (Cargill Animal Nutrition, Blair, NE) on a DM basis. Feed bunks in each pen were evaluated as required to allow for daily feed delivery adequate to ensure all calves had ad libitum access to feed without an excess of unconsumed feed accumulating in the feed bunk from 0 to 6 d. From 7 d to the end of the study (63 d) animals were fed according to bodyweight. Feeding was adjusting weekly based on the average animal bodyweight within the pen. Calves were fed once daily per normal procedures at the study site.

Approximately 12–24 h postarrival, calves were vaccinated with a killed vaccine against *Clostridium chauvoei, Clostridium septicum, Clostridium novyi, Clostridium haemolyticum, Clostridium perfringens* Types C and D and *Clostridium tetani* Calvary 9 (Calvary 9; Merck Animal Health, Madison, NJ) and a modified-live vaccine against infectious bovine rhinotracheitis (IBR), bovine viral diarrhea types 1 and 2 (BVDI-II), parainfluenza 3 (PI3), bovine respiratory syncytial virus (BRSV), and aid in the reduction and severity of pneumonic pasteurellosis due to *Mannheimia haemolytica* (Vista Once SQ; Merck Animal Health, Madison, NJ). Calves were treated for internal parasites with 10% Fenbendazole (Safe-Guard, Merck Animal Health) administered at a dose of 5 mg/kg of BW. Implants (Ralgro; Merck Animal Health) were administered upon the initiation of this study in the right ear of each calf. All animals were revaccinated on d 14 (Vista Once SQ; Merck Animal Health).

Calves received as bulls were castrated at processing in accordance with standard industry practice. Briefly, the scrotum was cleaned and disinfected using a cloth towel saturated with dilute iodine. The skin was surgically incised using a sharp, disinfected Newberry knife. The testes and spermatic cords were then exteriorized by blunt dissection. Testicles were pulled and the tunica, fascia, and blood vessels were stripped back as the testicles were pulled and removed.

Calves were blocked by sex upon arrival (bulls on arrival or steers on arrival) to ensure equal distribution of bulls and steers within each pen and by body weight (BW), and then randomly assigned to one of two study treatments with 16 pens/treatment. The two treatments were: 1) Calves received tildipirosin at 4 mg/kg subcutaneously only (PLBO) 2) Calves received tildipirosin at 4 mg/kg in combination with flunixin meglumine transdermal at a target of 3.3 mg/kg (FTD).

Tildipirosin was administered as a single subcutaneous injection. Flunixin transdermal was administered at a target of 3.3 mg/kg with a mean dose of 3.45 mg/kg bodyweight (equivalent to 1 mL/15 kg bodyweight), ranging from 3.21 to 3.84 mg/kg. The volume dosed per calf was determined using the dosing gauge on the product packaging. Due to this method of dosing, calves in the FTD group were given a mean (±SEM) 25.47 ± 1.6 additional milligrams of flunixin over their weight determined dose. A placebo was administered at the dose rate of 1 mL/15 kg in a similar manner as the flunixin transdermal. The placebo was made up of propylene glycol, isopropyl alcohol, and a red dye to mimic the test product in color, viscosity, and odor, as described in Kleinhenz et al. (2017). The flunixin transdermal and placebo were administered on dry skin. The entire dose was applied on the dorsal midline between the withers and tail head in accordance with label directions.

### Measurements and Sample Collection

Outcome variables measured for the entire 63 d feeding period were individual animal weights, pen weights, daily feed delivered, morbidity, and mortality. Additionally, visual analog scale (VAS) scores for pain assessment were obtained for the first 6 d. Accelerometer data were collected on a portion of the animals for the first 14 d to record animal activity. Individual animal weights were recorded on d 0, 14, and 63, and pen weights were determined by summing and averaging individual weights on these days.

Animals were observed twice daily for signs of morbidity that included overall depression, nasal and/or ocular discharge and anorexia. Any animal showing these signs was removed from the pen and taken to the hospital facilities where rectal temperature and a clinical illness score (CIS) were recorded. CIS were assigned as follows 1) normal healthy animal, 2) slightly ill with mild depression or gauntness, 3) moderately ill demonstrating severe depression/labored breathing/nasal or ocular discharge, and 4) severely ill and near death showing minimal response to human approach. Animals removed from the pen with a rectal temperature ≥ 40 °C and demonstrating a CIS ≥ 2 were treated following label instructions with the following compounds; at first morbidity animals received florfenicol (Nuflor: Merck Animal Health) administered as a single subcutaneous injection at a dose of 40 mg/kg BW; enrofloxacin (Baytril 100: Bayer Animal Health, Shawnee, KS) was administered at second morbidity at 12.5 mg/kg subcutaneously; Oxytetracycline (300 PRO LA; Norbrook Animal Health, Overland Park, KS) was administered subcutaneously at 30 mg/kg bodyweight as a single dose on the third treatment. Following the third treatment, the animal was considered chronic and removed from the trial. A BRD post-metaphylaxis interval (PMI) of 3 d after the use of tildipirosin on arrival, and a BRD posttreatment interval (PTI) of 3 d after the use of both florfenicol and enrofloxacin was observed.

A daily VAS pain assessment was conducted on three calves received as steers and three calves received as bulls and castrated on arrival, per pen, by two trained evaluators blinded to treatment allocations. Calves were chosen using the RAND function in Microsoft Excel (Microsoft Excel 2016, Microsoft Corporation, Redmond, WA). The VAS used was a 100 mm (10 cm) line anchored at each end by descriptors of “no pain” or “severe pain.” Five parameters were used to assess pain: depression, tail swishing or flicking, stance, head carriage, and foot stomping or kicking. No pain was characterized by being alert and quick to show interest, no tail swishing, a normal stance, head held above spine level, and absence of foot stomping. Severe pain was characterized by being dull and showing no interest, more than three tail swishes per minute, legs abducted, head held below spine level, and numerous stomps. The evaluator marked the line between the two descriptors to indicate the pain intensity. A millimeter scale was used to measure the score from the zero anchor point to the evaluator’s mark. VAS assessments were taken every 12 h, starting 12 h after being processed onto the study and continuing for 6 d. The mean VAS measures of the two evaluators were combined into one score for statistical analysis.

IceTag (IceRobotics Ltd, South Queensferry, Edinburgh, UK) accelerometers were placed on 40 animals (10 per study lot × 4 lots) on the day of enrollment on at least one calf per pen. Calves were chosen using the RAND function in Microsoft Excel (Microsoft Excel 2016, Microsoft Corporation, Redmond, WA). Accelerometers were placed on the left rear legs. Accelerometer ID was paired with calf ID before placement onto the calf. Accelerometers were removed and collected at the time of revaccination (14 d) and returned to Dr. Ty Lawrence at West Texas A&M University for data download. Raw data were returned to study investigators for analysis. Steps, standing up, lying down and lying bouts, and motion index data were collected via accelerometers.

The motion index, steps, and lying bouts were summed into 12 h increments starting at 0600 h of d 1 and ending at 0600 of d 14 for 28–12 h intervals. Standing time and lying time were analyzed together due to their interrelation and were summed on a 24 h increment to account for the recording method of the accelerometer. Step counts and motion index for each 12 h increment were log transformed for normality.

### Calculations and Statistical Analysis

Statistical analysis was performed using computer software (JMP 13, SAS Institute, Cary, NC). Responses were analyzed using a mixed linear model with pen as the experimental unit using AR-1 as the covariance structure. Pen nested in a treatment group (FTD or PLBO) and lot were designated as a random effect with treatment, time (DOF), and treatment by time interaction as fixed effects. Pair-wise comparisons were done using Tukey-HDS tests. Responses measured included initial BW, interim BWs, final BWs, gain, average daily gain (ADG), gain to feed, dry matter intake (DMI), morbidity, mortality, case fatality, removals, accelerometer activity data, VAS measures, and number of cattle that were pulled but not treated. BW was determined by weighing individual animals; ADG was calculated as initial BW—final BW / total days on feed; DMI was calculated as daily pen feed allocation—feed remaining at next feeding / number of calves in the pen; gain to feed was calculated as pen level ADG / pen level DMI. Statistical significance was set a priori at *P* ≤ 0.05.

## RESULTS

### Performance

**Figure 1.**
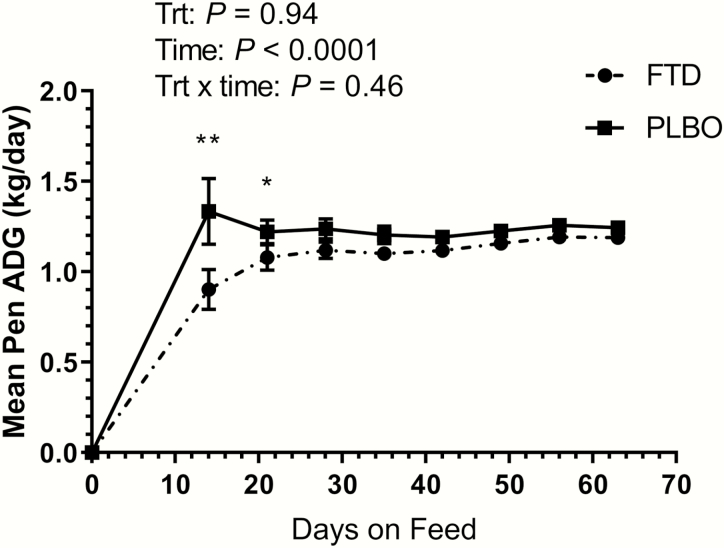
Mean pen ADG for calves treated with tildipirosin in combination with transdermal flunixin (FTD) or tildipirosin alone (PLBO) at arrival. **P ≤ 0.05. *P ≤ 0.10.

**Figure 2.**
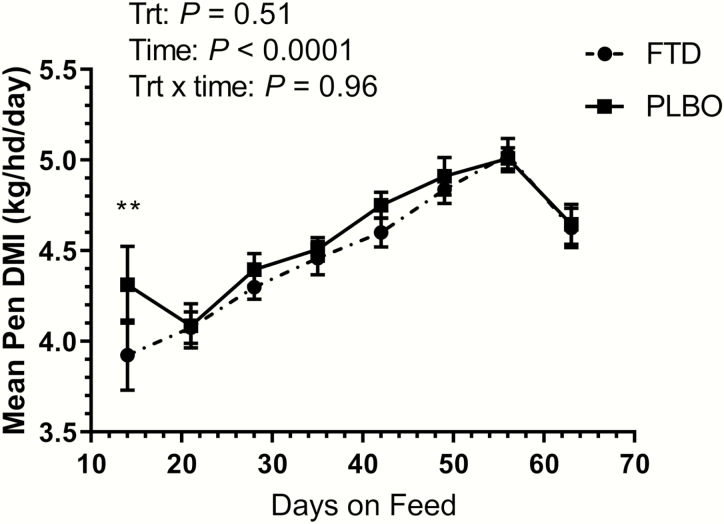
Mean pen DMI for calves treated with tildipirosin in combination with transdermal flunixin (FTD) or tildipirosin alone (PLBO) at arrival. **P ≤ 0.05.

Performance data were calculated with data from the cattle that died or were removed from the study because of medical conditions that occurred during the 63 d feeding period excluded. During the 63-d feeding period, performance was not affected by treatment (Table 1); FTD calves had similar ADG and dry matter intake (DMI) to PLBO calves over the 63-d feeding period (*P* = 0.94 and *P* = 0.51, respectively). However, during the first 14 d on feed, calves treated with FTD had a lower ADG of 0.90 kg/d compared with 1.33 kg/d in the PLBO group (*P* = 0.05; Figure 1). During the first 14 d on feed, calves treated with FTD also demonstrated a lower overall weight gain of 12.6 kg compared with 18.7 kg in the PLBO group (*P* = 0.04; Figure 1). On d 14, the DMI for the FTD group was 3.92 kg/hd/d compared with 4.31 kg/hd/d (*P* = 0.01) in the PLBO group (Figure 2). Additionally, there was a significant effect of days on feed for DMI (*P* < 0.001).

**Table 1.** Mean performance summary in kilograms for calves treated with tildipirosin in combination with transdermal flunixin (FTD) or tildipirosin alone (PLBO) at arrival

	Period	FTD	PLBO		
Item	d	kg	kg	SEM	*P* value
Number of pens		16	16		
Number of bulls		100	99		
Number of steers		92	93		
BW	0	221.7	221.3	3.52	0.95
BW	14	234.3	240.0	3.52	0.42
BW	63	296.7	299.7	3.52	0.60
BW gain	0–14	12.6^a^	18.7^b^	1.99	0.04
BW gain	14–63	62.4	59.6	2.26	0.39
BW gain	0–63	75.0	78.4	1.73	0.18
ADG	0–14	0.90^a^	1.33^b^	0.15	0.05
ADG	14–63	1.29	1.14	0.10	0.30
ADG	0–63	1.10	1.10	0.05	0.94
DMI	0–14	3.92^a^	4.31^b^	0.13	0.01
DMI	14–63	4.59	4.61	0.08	0.55
DMI	0–63	5.70	5.77	0.08	0.51
G:F	0–14	0.23	0.31	0.04	0.18
G:F	0–63	0.27	0.27	0.01	0.69

G:F, gain to feed ratio.

^a,b^Performance within days on trial with different superscripts are significantly different (*P* ≤ 0.05).

### Health

The health data are summarized in Table 2. During the 63-d feeding period, health parameters were not affected by treatment. There were 168 calves identified for health evaluations over the course of the study and 118 calves treated for at least one treatment regimen, resulting in 30.73% overall morbidity. There was no effect of treatment on mean days to first pull (*P* = 0.64) or mean days to first treatment (*P* = 0.29). The overall chronic removal rate was 3.91% with 66.67% of the chronic removals due to BRD. The overall mortality rate was 4.95% with 89.47% of the mortalities due to BRD. One calf from each treatment group died as a result of the castration procedure (exsanguination).

**Table 2.** Health summary for calves treated with tildipirosin in combination with transdermal flunixin (FTD) or tildipirosin alone (PLBO) at arrival

	FTD	FTD	FTD	PLBO	PLBO	PLBO
Item	*n*	%	d	*n*	%	d
Total morbidity		33.3			27.6	
BRD first treatment	61	31.8		51	26.6	
BRD second treatment	25	13.0		21	10.9	
BRD third (chronic)	4	2.1		6	3.1	
First treatment success rate		56.3			58.8	
BRD observations not treated		14.6			12.0	
Days to first BRD pull			12.4			11.9
Treated for lameness	4	2.1		3	1.6	
Other treatment	1	0.5		2	1.0	
BRD mortality	10	5.2		7	3.6	
Non-BRD mortality	1	0.5		1	0.5	
Overall mortality	11	5.7		8	4.2	
BRD removal	4	2.1		6	3.1	
Non-BRD removal	2	1.0		3	1.6	
Overall removal	6	3.1		9	4.7	

There were no significant differences between treatment groups (*P* ≤ 0.05).

### VAS Pain Assessment

**Figure 3.**
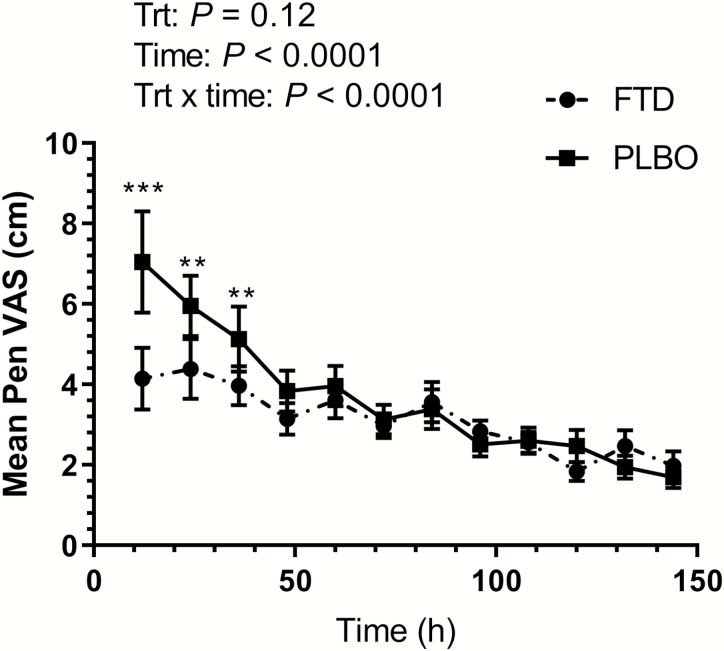
Mean pen VAS assessment over time for calves treated with tildipirosin in combination with transdermal flunixin (FTD) or tildipirosin alone (PLBO) at arrival. ***P < 0.001. **P ≤ 0.05.

VAS pain assessment data are presented in Figure 3. There was a significant time by treatment interaction (*P* < 0.001). During the first 36 h after treatment administration, the FTD-treated calves had lower VAS measures [6.23 (95% CI: 5.27–7.20) compared with 7.28 (95% CI: 6.32–8.24)] than PLBO (*P* ≤ 0.05). VAS measures for both groups decreased over time.

### Accelerometer Activity Data

The standing time, lying time, steps, and lying bouts of two animals were excluded from analysis as the accelerometer failed to record data. Accelerometer data are summarized in Table 3. There was an increase in the motion index (amount of movement) in the first 12 h and this is shown as a significant time effect (*P* < 0.001). After the first 12 h, the motion index had a diurnal pattern, but there were no differences in the motion index between treatment groups (*P* = 0.94). Similar to motion index, both treatment groups had an increased number of steps in the first 12 h, then the number of steps ranged between 250 and 597 steps per 12-h interval. There was no effect of treatment or treatment by time interaction for the number of steps. There was a significant time effect (*P* = 0.01) on number of lying bouts but there were no significant treatment effects (*P* = 0.19) or treatment by time interactions (*P* = 0.68). There were no differences in the amount of time standing or lying for each treatment group (*P* = 0.86) and no interaction with time (*P* = 0.99).

**Table 3.** Accelerometer summary for calves treated with tildipirosin in combination with transdermal flunixin (FTD) or tildipirosin alone (PLBO) at arrival

Item	FTD	PLBO	SEM	*P* value
Motion index	1,749	1,814	112	0.96
Standing, min	603.4	609.8	12.96	0.78
Lying, min	841.5	834.8	12.96	0.72
Lying, bouts	8.44	7.00	0.52	0.08
Steps	422.1	396.1	13.2	0.25

There were no significant differences between treatment groups (*P* ≤ 0.05).

## DISCUSSION

In the present study, the topical administration of flunixin meglumine transdermal in combination with tildipirosin did not significantly improve receiving performance compared with cattle that only received tildipirosin. These results are consistent with the findings of Cooke et al. (2013), who observed that injectable flunixin meglumine did not improve performance of feeder cattle. Gifford et al. (2012) observed that with a greater incidence of clinical signs of BRD, comes a decrease in ADG and BW. In the present study, no significant differences were observed in health measurements between treatment groups. Conversely, failure to decrease clinical signs of BRD may be attributable to the lack of significant differences in performance between treatment groups.

Although ADG and DMI were similar over the 63-d feeding period, calves who received flunixin meglumine had lower ADG and DMI on d 14. González et al. (2010) observed a reduction in intake but not ADG after flunixin meglumine and xylazine co-administration following band castration over a 6-wk period. However, Coetzee et al. (2012) did not observe any effect of meloxicam administration on ADG or DMI of surgically castrated calves over a 28-d period, indicating that a reduction in intake may be specific to certain NSAIDs and not others. Whether or not this effect is profound enough to be biologically relevant should be considered.

There were no observed differences in morbidity or mortality due to BRD between the two treatment groups. However, since both treatment groups received tildipirosin, it may have reduced the incidence of BRD from what it would have been without metaphylaxis. Tildipirosin has been observed to lower the hazard of being affected with BRD and/or otitis (Teixeira et al., 2017) and has been shown to be more effective than tilmicosin at lowering first-pull treatment rates for BRD (Donkersgoed and Merrill, 2013). Since the calves in the present study were considered high-risk, metaphylaxis was used throughout the study in the interest of animal well-being.

Pain is defined as an aversive sensory experience associated with actual or potential tissue damage; it results in physiologic, neuroendocrine, and behavioral changes that indicate a stress response in the animal (Molony and Kent, 1997). Postoperative inflammatory pain should be treated by using an NSAID (Huber et al., 2013). In the present study, visual analog assessment results indicated that calves coadministered flunixin transdermal and tildipirosin showed less signs of pain the first 36 h postdrug application. Several types of pain responses can be recognized: 1) those that modify the animal’s behavior to avoid the reoccurrence of the experience; 2) those that protect the animal such as withdrawal reflexes; 3) those that minimize pain and assist with healing such as lying or standing still; 4) those that elicit help or to stop another animal or human from inflicting more pain such as vocalization or posture (Molony and Kent, 1997). Our results indicate that the administration of an NSAID postoperatively reduced visual signs of pain in calves up until 36 h after administration. Kleinhenz et al. (2018) observed that administration of transdermal flunixin reduced plasma cortisol concentration and mitigated the stress response in calves for 8 h when given at the time of castration. However, negligible effects on the pain biomarkers of substance P, ocular infrared thermography, and gait analysis were observed (Kleinhenz et al., 2018). NSAIDs alone are not effective in reducing the acute distress associated with castration, but their analgesic and anti-inflammatory effects do extend into the postoperative period (Coetzee, 2013).

Assessing pain by monitoring animal behavior can be difficult and often subjective. One means of measuring changes in behavior outcomes without the presence of human evaluators is through accelerometers—which reduce subjectivity and outside influence (White et al., 2008). Currah et al. (2009) observed that stride length and the number of steps taken by calves after castration can be good measures of pain, with castrated calves taking fewer steps and showing less activity. Calves treated with flunixin were observed to have decreased stride lengths after drug application, but significantly longer stride lengths 4 and 8 h postdrug application compared with calves not administered flunixin (Currah et al., 2009). It has also been observed that calves spent significantly more time standing after castration than before castration (White et al., 2008). In the present study, both treatment groups were observed to have an increased number of steps in the first 12 h when compared with the following 13 d. No significant differences were observed between treatments in the amount of time standing, time lying, steps, motion index, or lying bouts. Accelerometer activity data seems to be a promising way to more objectively evaluate animal behavior without human intervention and warrants further investigation.

## CONCLUSION

Results of this study suggest that there were no significant advantages in performance, health, or activity measured by accelerometers from the co-administration of flunixin meglumine transdermal and tildipirosin. However, calves coadministered flunixin meglumine transdermal and tildipirosin did have lower visual analog scale scores indicating that less pain was apparent the first 36 h postdrug application relative to calves only administered tildipirosin.
